# Computed tomography pulmonary angiography (CTPA) for the detection of pulmonary embolism (PE) among trauma patients: a systematic review and meta-analysis

**DOI:** 10.1007/s10140-024-02249-7

**Published:** 2024-06-07

**Authors:** Shirin Yaghoobpoor, Mobina Fathi, Hayder Jasim Taher, Afraa Jasim Farhood, Ashkan Bahrami, Reza Eshraghi, Ramtin Hajibeygi, Zohreh Tutunchian, Lee Myers, Rojin Ahmadi, Ali Gholamrezanezhad

**Affiliations:** 1 Advanced Diagnostic and Interventional Radiology Research Center (ADIR), Tehran, Iran; 2 Student Research Committee, Faculty of Medicine, Shahid Beheshti University of Medical Sciences, Tehran, Iran; 3 Department of Radiology, Hilla University College, Babylon, Iraq; 4 Imam AL sadiq teaching hospital / Babylon Health director, Babylon, Iraq; 5 Faculty of Medicine, Kashan University of Medical Science, Kashan, Iran; 6 Department of Radiology, University of Southern California, Keck School of Medicine, Los Angeles, California USA; 7 School of Medicine, Islamic Azad University Medical Branch of Tehran, Tehran, Iran; 8 Department of Radiology, Keck School of Medicine, University of Southern California (USC), Los Angeles, USA

**Keywords:** Pulmonary embolism, Trauma patients, Computed tomography pulmonary angiography, Prevalence, Systematic review, Meta-analysis

## Abstract

**Background and objectives:**

Computed tomography pulmonary angiography (CTPA) is a standard imaging technique employed for the detection of pulmonary embolism (PE). This systematic review and meta-analysis aims to examine the prevalence of PE among the trauma patients undergoing CTPA.

**Methods:**

A comprehensive search across PubMed, Scopus, Google Scholar, and Web of Science yielded 13 studies encompassing 5,570 individuals conducted following Preferred Reporting Items for Systematic Review and Meta-Analysis (PRISMA) guideline. Studies that used CTPA for the detection of PE among the trauma patients were selected. This resulted in an evaluation of prevalence, trauma types, clinical manifestations, radiological findings, and mortality rates of PE among traumatic patients undergoing CTPA.

**Results:**

The overall prevalence of PE among trauma patients undergoing CTPA was 18% (95% CI = 13-24%). After pooling the existing data, femur fractures were determined to be the most prevalent trauma type (12%). The most prevalent clinical manifestations of PE among trauma patients included shortness of breath, chest pain, and altered vital signs. Radiological findings encompassed various pulmonary abnormalities, such as opacity, ground-glass opacities, and pleural effusions. Mortality rates of PE among the trauma patients ranged from 0% to 29.4% across the included studies.

**Conclusion:**

This study provides comprehensive insights into the prevalence, clinical manifestations, radiological findings and mortality of PE among trauma patients undergoing CTPA. According to our findings, lower threshold for CTPA is recommended in patients with lower extremity or spine fractures.

## Introduction

Pulmonary embolism (PE) is a life-threatening respiratory disorder in the setting of trauma and has been on the rise in recent decades. PE can lead to increased alveolar dead space and hypoxemia, increasing pulmonary vascular resistance and subsequent right heart failure. In acute setting, pulmonary thromboembolism can develop from inflammation, endothelial injury and the hypercoagulable state related to the traumatic event [1]. PE can also arise from immobilization during the post-traumatic hospital course with a high percentage of PEs occurring in the first 72 hours after injury [4]. PE is a potentially preventable hospital mortality in the setting of trauma and appropriate strategies should be undertaken to ensure early diagnosis.

There are several strategies for diagnosing PE such as using clinical prediction scores including the Wells score and Geneva score. Traumatic injuries may cause elevated D-dimer levels independent of PE in the acute phase of injury [2–4]. Additionally, the classic PE signs and symptoms including leg swelling, dyspnea, tachypnea and hypoxemia can be masked by traumatic injuries. Computed tomographic pulmonary angiography (CTPA) is the gold standard for PE diagnosis. CTPA is a fast, diagnostic, reliable, and widely available imaging method. These attributes have resulted in a significant increase in the number of trauma patients undergoing imaging for diagnosing PE since the 1990s [5–7].

Buchanan et al. found that clinical variables and current risk scoring models do not differentiate patients with and without PE in trauma patients [11]. Cerbasi et al. found that patients with pelvic trauma should undergo lower extremity doppler ultrasound to evaluate for deep vein thrombosis (DVT) before and sixteen days after surgery [12]. Gudipati et al. reviewed 18,151 trauma patients with orthopedic procedures, 85 of which developed PE (61 acute trauma and 24 underwent elective surgery). Mortality rate in those that developed PE was 15.29% [4]. The current systemic review and meta-analysis was conducted to evaluate the detection power of CTPA for PE among trauma patients and assessing clinical manifestations, mortality rates, and common radiological findings.

## Materials and methods

### Study selection

The study adhered to the Preferred Reporting Items for Systematic Review and Meta-Analysis (PRISMA) guidelines to conduct a thorough systematic search. A comprehensive search was performed on credible databases (PubMed, Scopus, Google Scholar, and Web of Science) using a specified search query that included key terms; “Computed Tomography Pulmonary Angiography”, “Pulmonary Embolism”, “Trauma Patients”. Finally, 13 articles published before March 2024 were collected in the current study. The references to each publication were thoroughly reviewed to ensure no study is missed.

### Inclusion and exclusion criteria

We selected 13 articles that performed CTPA for trauma patients and reported prevalence of diagnosed PE among them. The articles underwent a comprehensive review and examination focusing on their setting, design, scope, and data. Studies that examined radiographic modalities other than CTPA, non-traumatic cases, non-English articles, case reports, review articles, and those with insufficient data were excluded.

### Data extraction

To ensure accuracy and reliability, data extraction was performed by two independent authors (A.B and S.Y), who carefully reviewed and examined the final set of publications. They collected data on various parameters, including the number of patients, sex, age, mechanism of injury, death rate, length of hospital stays, clinical manifestations, radiological findings of CTPA, and number of PE cases.

### Statistical analysis

A meta-analysis was conducted using Stata version 15 USA to calculate the pooled prevalence of PE among trauma cases which underwent CTPA. The prevalence rate was chosen as the unit for effect magnitude. Data was analyzed using a random effect model. I^2^ statistics were utilized to evaluate heterogeneity, with values exceeding 50% indicating substantial heterogeneity. Publication bias was assessed quantitatively by Begg’s and Egger’s test and illustrated with funnel diagrams. Due to insufficient data, no subgroup analysis or meta-regression analysis was applicable.

### Publication bias

**Fig. 1:**
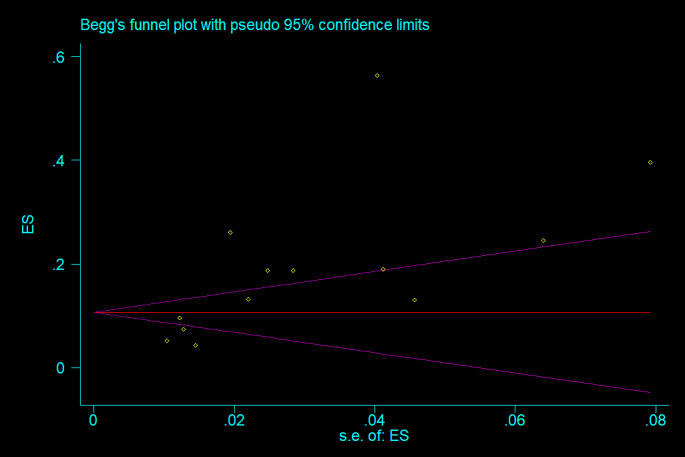
The Begg’s test diagram showing the publication bias among the included studies

The Egger’s and Begg’s test evaluated publication bias in the selected literature. A significant publication bias is indicated by *P* < 0.05 (Fig. 1). Linear regression analysis was conducted with intercept and slope parameters. The formula used to determine the parameters was yi 1/4 a + bxi + ϵi, where yi represents the standardized estimate, xi represents the precision of studies, and ϵi represents the error terms, with i ranging from 1 to r (r being the number of studies).

### Quality assessment

The quality of each included paper was assessed and documented using the Newcastle-Ottawa Scale (NOS) [8]. The NOS checklist for cross-sectional studies uses 8 distinct evaluations, such as ‘selection’, ‘comparability’, and ‘result’, to assign a correlational score reflecting each study’s statistical power. The NOS grading method for cross-sectional studies is outlined below: Very good studies (9–10 points), Good studies (7–8 points), Satisfactory studies (5–6 points), and Unsatisfactory studies (0 to 4 points).

## Results

### Study selection and characteristics

**Fig. 2:**
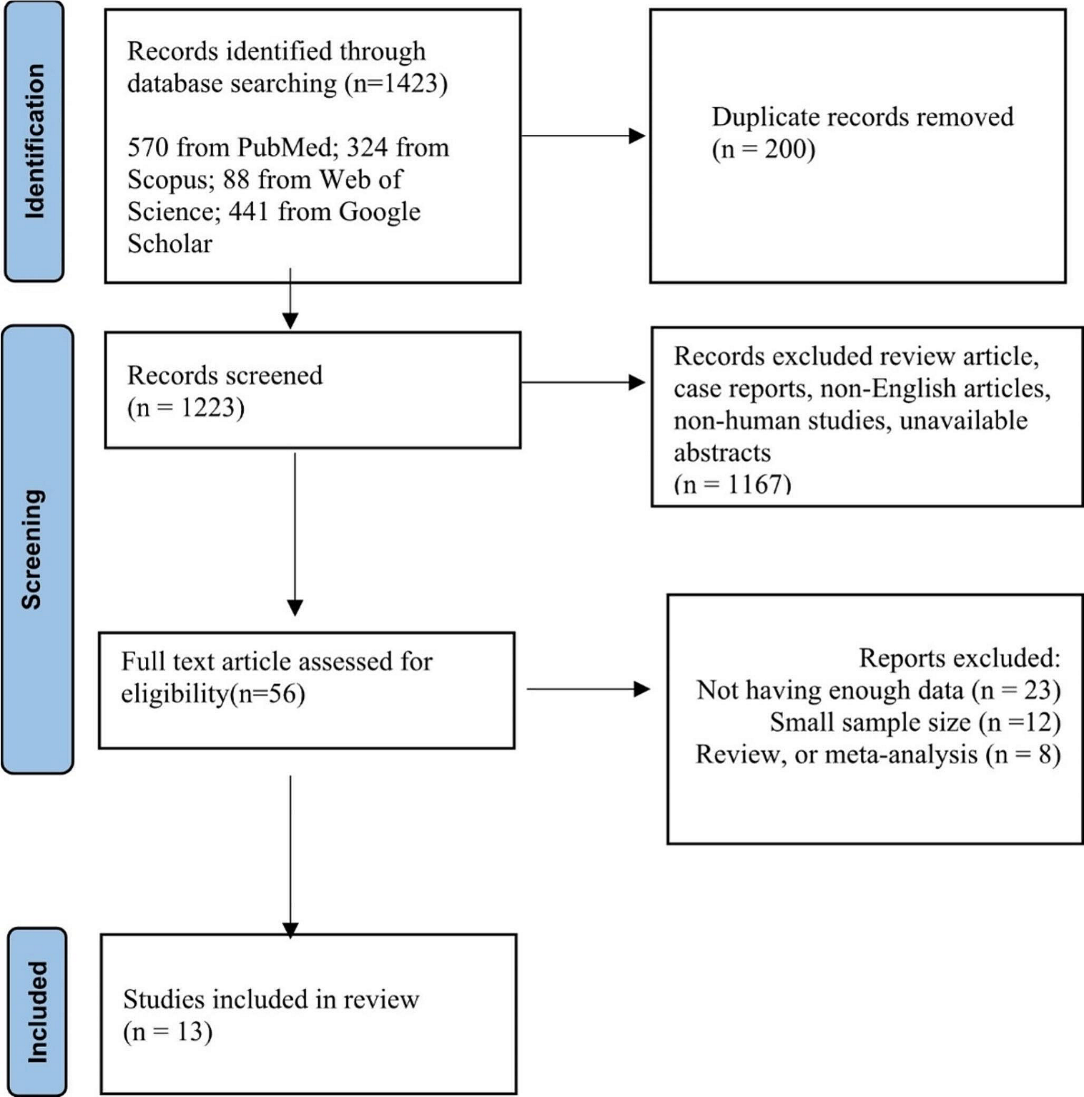
Study flow diagram

This study was performed following the PRISMA guidelines. A total of 1423 articles were found through the initial search of databases including PubMed (570), Scopus (324), Google Scholar (441), and Web of Science (88). After removing duplicates, 1223 articles remained. After title and abstract screening, 1167 articles were excluded because of being non-English, not performing CTPA, or irrelevant to trauma. After full text screening 43 articles were excluded due to small sample size (*n* = 12), review or meta-analysis (*n* = 8), or not reporting sufficient data (*n* = 23). Finally, 13 articles were included in our systematic review and meta-analysis (Fig. 2).

13 cohort studies (9 retrospective [4, 9–16], 3 prospective [17–19]), and one cohort study [20] were included in this study. The total sample size in studies that have mentioned, was 5570 individuals. A total of 8794 trauma patients undergoing CTPA were included in our analysis. The mean age of the total sample size in studies that have mentioned, was 57.17. Males contributed 61% of the total population (Table 1).

**Table 1:** Baseline characteristics in studies included in this meta-analysis

Author	Year	Country	Study type	Sample size	Age(mean)	Gender (male)	Trauma type	Trauma number	Patients with CTPA	Age(mean) of PE or DVT	Gender (male) of PE or DVT	PE Number	DVT	Mortality
Buchanan et al. [9]	2021	USA	Retrospective Cohort	15,861				*n* = 15,861	*n* = 235	52	*n* = 21 (67%)	*n* = 31		
Cerbasi et al. [17].	2022	Italy	Prospective study	191	49.6	*n* = 148 (77.5)	Acetabular fractures, Pelvic fractures	*n* = 191	*n* = 191			All: *n* = 8, All fractures: *n* = 8 of 191 (4.2%), Acetabular fractures: *n* = 4 of 118 (3.4%), Simple: *n* = 2 of 71 (2.8%), Associated: *n* = 2 of 47 (4.3%), Pelvic fractures: *n* = 4 of 73 (5.5%)		
Costantino et al. [10]	2008	USA	Retrospective Cohort	575				*n* = 575	*n* = 575			*n* = 55 (8.6%)		
Dwivedi et al. [11]	2022	Australia	Retrospective Cohort	38				*n* = 38	*n* = 38	31.2	*n* = 12 (80%)	FES: *n* = 15		
Gudipati et al. [12].	2014	UK	Retrospective Cohort	18,151			Trauma operation (orthopedic surgery)	*n* = 7503	*n* = 5656	66.3	*n* = 42 (49.4%)	All: *n* = 85, Trauma: *n* = 61 (71.76%), Elective orthopedics surgery: *n* = 24 (28.23%)		*n* = 13 (15.3%)
Kim et al. [4].	2017	Republic of Korea.	Retrospective Cohort	446	78	*n* = 135 (30.3%)	Femur fracture	*n* = 446	*n* = 446	79	*n* = 10 (43.5%)	*n* = 23		*n* = 0
Licht et al. [13].	2008	Israel	Retrospective Cohort	4559	56	*n* = 2833 (62%)		*n* = 54 of Late Scan group (CT scans performed after 24 h in the ICU: *n* = 113), *n* = 10 of the Admission Scan group (performed from 24 h prior to 24 h post ICU admission: *n* = 51), all: *n* = 64	*n* = 164	Late Scan group (CT scans performed after 24 h in the ICU: *n* = 113) PE positive: 45/ Admission Scan group (performed from 24 h prior to 24 h post ICU admission: *n* = 51): 67	All: *n* = 14 of 21 (66.67%), Late Scan group (CT scans performed after 24 h in the ICU: *n* = 113) PE positive : *n* = 6 (86%), Admission Scan group (performed from 24 h prior to 24 h post ICU admission: *n* = 51): *n* = 8 (57%)	All: *n* = 21, Late Scan group (CT scans performed after 24 h in the ICU: *n* = 113) PE positive *n* = 7 of 113 (6%), Admission Scan group (performed from 24 h prior to 24 h post ICU admission: *n* = 51): 14 of 51 (27%)		Late Scan group (CT scans performed after 24 hours in the ICU: *n* = 113) PE positive(ICU Mortality) *n* = 1 of 7 (14%)/ Admission Scan group (performed from 24 hours prior to 24 hours post ICU admission: *n* = 51)PE positive(ICU Mortality): 2 of 14 (14%)/ all PE positives: *n* = 3
Malinoski et al. [18]	2013	USA	Prospective cohort	411	48	*n* = 296 (72%)	Trauma, Blunt	All: *n* = 411, Blunt Trauma (In patients with LE DVT or PE: ): *n* = 27 (90%)	*n* = 411	45.6	*n* = 24 (80%)	*n* = 30		
McDuffie et al. [14]	2022	USA	Retrospective Cohort	14,968			Severe Lower Extremity Injury, Severe Abdominal Injury, Severe Thorax Injury, Severe Head Injury, Major Vascular Injury, Pelvic Fracture, Spinal Cord Injury, Major Surgery	All: *n* = 508, Severe Lower Extremity Injury *n* = 49 (37.12%), Severe Abdominal Injury *n* = 32 (24.24%), Severe Thorax Injury *n* = 58 (43.94), Severe Head Injury *n* = 32 (24.24%), Major Vascular Injury *n* = 4 (3.03%), Pelvic Fracture *n* = 31 (23.48%), Spinal Cord Injury *n* = 5 (3.79%), Major Surgery *n* = 117 (88.64%)	*n* = 508	46.8	*n* = 99 (75%)	*n* = 132	DVT: *n* = 131, DVT with PE: *n* = 21	*n* = 5 (3.3%)
Minshall et al. [15]	2015	USA	Retrospective Cohort	188				*n* = 188	*n* = 188	40.7		*n* = 35 (18%)		*n* = 6 (17%)
Tofigh et al. [20].	2011	Iran	Cohort	45	38	*n* = 36 (80%)	Motor accident, Stab wound, Bullet///Concurrent: bone trauma: Supracondylar fracture, Posterior knee dislocation, Multi-fragment knee fracture, Tibial plateau fracture, Vascular trauma Venous, Arterial, and venous	All: *n* = 45, Motor accident: *n* = 34 (76%), Stab wound: *n* = 6 (13%), Bullet: *n* = 5 (11%)///Concurrent: bone trauma: Supracondylar fracture: *n* = 10 (22%), Posterior knee dislocation: *n* = 8 (18%), Multi fragment knee fracture: *n* = 7 (16%), Tibial plateau fracture: *n* = 5 (11%), Vascular trauma Venous: *n* = 15 (33%), Arterial and venous: *n* = 30 (67%)	*n* = 45			*n* = 11 (26%)		*n* = 2 (4%)
Velmahos et al. [16]	2009	USA	Retrospective Cohort	247			Trauma, Blunt	All: *n* = 247, PE With DVT: *n* = 7 of 7 (100%), PE Without DVT: *n* = 36 of 39 (92%)	*n* = 247	PE With DVT: 56, PE Without DVT: 56	PE With DVT: *n* = 4 (57%), PE Without DVT: *n* = 25 (64%)	all: *n* = 46/ PE With DVT: *n* = 7 / PE Without: DVT *n* = 39	*n* = 18 (7%)	*n* = 2 (4.34%)
Zhou et al. [19]	2021	China	Prospective cohort	90	81.2	*n* = 31 (34.4%)	Hip fracture: Femoral neck fracture, Intertrochanteric fracture	Femoral neck fracture: *n* = 40 of 90 (44.4%), Intertrochanteric fracture: *n* = 50 of 90 (55.6%)	*n* = 90	81.2	*n* = 10 (58.8%)	*n* = 17		30 days: *n* = 1 (5.88%), 90 days: *n* = 2 (11.76%), 1 year: *n* = 5 (29.41%)

### Prevalence of PE among trauma patients undergone CTPA

**Fig. 3:**
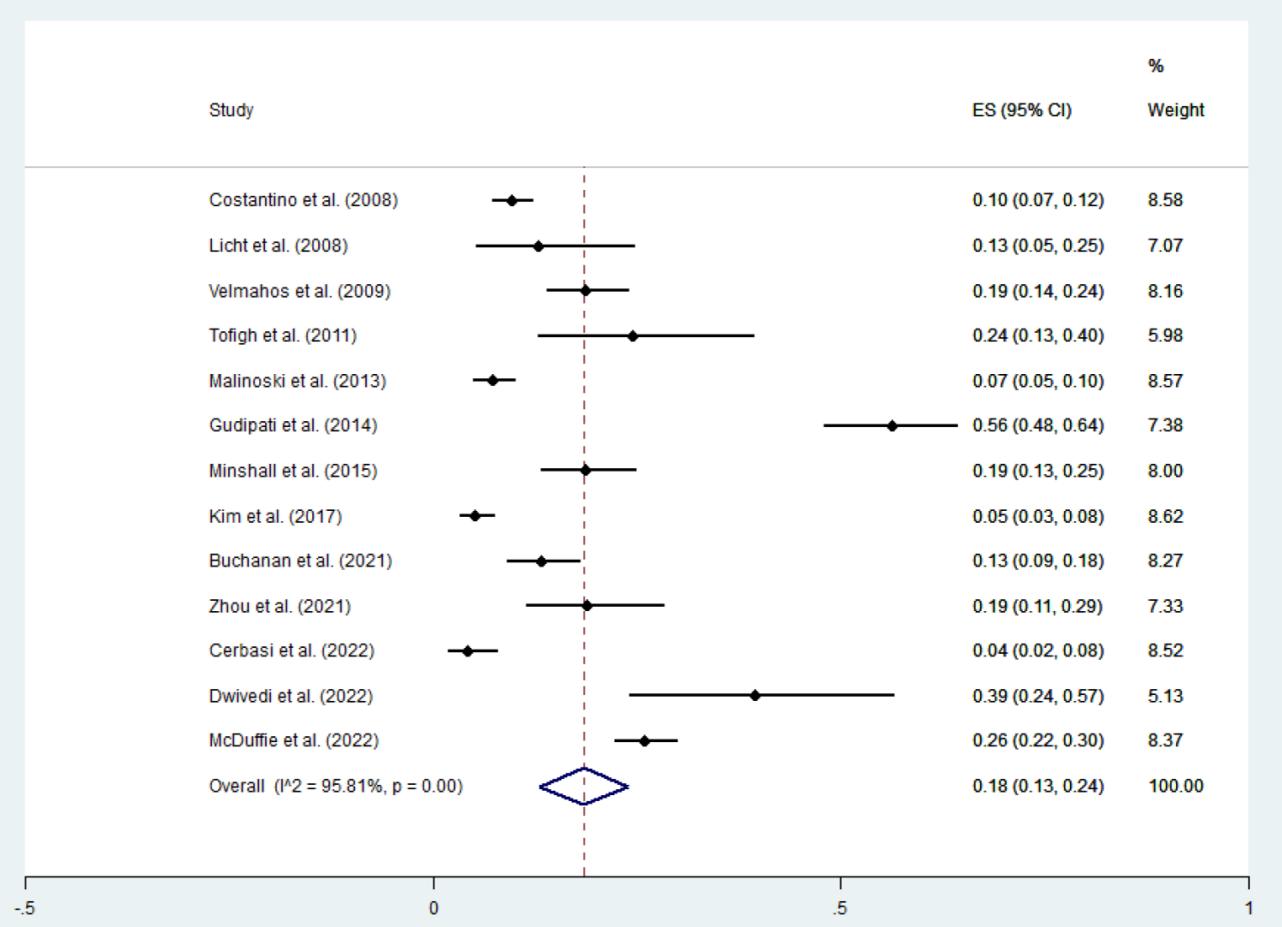
The forest plot indicating the pooled prevalence of PE among trauma patients undergone CTPA. The weight of each paper on the meta-analysis is indicated by each parallelogram, the 95% CI is visualized by the interval within the boundaries. Literature is presented based on random effect model.

According to our meta-analysis, the prevalence of PE among the trauma patients who had undergone CTPA was 18% (95%CI = 13-24%), however, the heterogeneity was high (95.81%). The random effects model appropriately accounts for the variability, and there was no significant publication bias detected. However, the substantial heterogeneity (I² = 95.81%) suggests significant variability among the studies; furthermore, due to insufficient data, no subgroup or meta-regression analysis was applicable to find the source of heterogeneity (Fig. 3).

### Trauma types

The most prevalent trauma among the included patients was femur fracture, reported in 693 cases (12%). Other reported types of traumas were spine fracture in 285 (4%), lower extremity fracture in 171 (excluding fractures of the femur) (2%), upper extremity fracture in 96 (1%), pelvic fracture in 82 (1%), thorax fractures in 58 (1%), vascular trauma in 49 (0.8%), head fracture in 32 (0.5%), abdominal trauma in 32 (0.5%), and spinal cord injury in 32 (0.5%). Others were not mentioned.

### PE among trauma patients who were evaluated with CTPA

In a study by Licht et al [13]. within 2008, 21 of 164 trauma patients (12.8%) had PE. Positive scan patients had a higher rate of head trauma (5/7 positive scans, 71% versus(vs). 23/106 negative scans, 22%, *P* = 0.005), spine injury with neurological impairment (4/7, 57% vs. 9/106, 8%, *P* = 0.002), and lower limb injury (3/7, 43% vs. 12/106, 9%, *P* = 0.039). DVT prophylaxis before a positive scan was less common (3/7, 43% vs. 91/106, 86% *P* = 0.015). On limited logistic regression, only recognized risk factors reliably predicted positive CT angiography (OR 24.7 per risk factor, 95% CI= 2.38 to 255.1, *P* = 0.007 ). Among the late scan group, which included patients who had CT scans after 24 hours in the intensive care unit (ICU) (n = 113), 7 out of 113 (6%) confirmed positive for PE. Conversely, among the admission acan group, whose included scans taken from 24 hours before to 24 hours prior ICU admission (n = 51), 14 out of 51 (27%) were determined to have PE [13]. In a study conducted by Constantino et al. [10] in 2008, it was determined that PE was diagnosed in 9.57% of 575 trauma patients who were evaluated with CTA [10]. In a study conducted in 2009 in the United States by Velmahos et al. [16] on 247 trauma cases with blunt or penetrating trauma, PE was investigated via CTPA imaging, among 247 trauma cases undergoing CTPA or computed tomography venography, 46 (19%) had PE and 18 (7%) had DVT only, 39 cases of 247 (15.8%) had PE only, and 7 of 247 (2.8%) had both PE and DVT [16]. In a 2011 study conducted in Iran, Tofighi et al. [20] on 45 patients with popliteal vein damage operated between 2006 and 2009, CT angiography revealed PE in 11 (26%) of the patients. Malinoski et al. [18]’ study in 2013 on trauma patients with CTPA, showed that 30 (7.3%) of 411 cases had venous thromboembolism (VTE), with 28 of 411 (6.8%) having lower extremity DVT and 2 of 411 (0.5%) having PE [18].

Gudipati et al. [12] conducted a study in 2014 on trauma and orthopedic patients who underwent CTPA evaluation. The findings showed that out of 5656 patients, 85 (1.5%) were diagnosed with PE. In 61 out of 85 patients diagnosed with PE (71.76%) trauma was the primary etiology; while, 24 out of 85 (28.23%) patients diagnosed with PE had undergone elective orthopedic surgery secondary to trauma [12]. In a 2015 retrospective study conducted in the United States by Minshall et al. [15] on trauma patients CTPA was used and 35 (18%) PE cases among the total 188 patients were reported. In a study by Kim et al. [4] in 2017, 446 adult patients with femur fractures and elevated D-dimer levels (> 0.5 g/mL) who got CTPA within 72 hours after trauma were included, in which, 23 patients (5.2%) were diagnosed with acute PE. The clinical likelihood of PE was determined using a modified Wells scale and revised Geneva ratings [4]. Buchanan et al. in 2021 [9] evaluated 235 trauma patients that had undergone CTPA in 2021, with only thirty-one trauma patients (13%) having clinical signs of PE [9]. In a study conducted in 2021 in China by Zhou et al. [19] on 90 trauma patients with hip fracture, CTPA was used to detect preoperative asymptomatic PE and indicated that 17 cases out of 90 (18.9%) had PE [19]. In a study by Cerbasi et al. [17] in 2022, postoperative DVT was seen in 42 of 191 patients (22%). Eight patients (4.2%) out of 191 had PEs, three (1.5%) of which were DVT-related [17]. Dwivedi et al. in 2022 [11] reported that 15 out of 38 patients were diagnosed with fat embolism syndrome detected on CTPA, accounting for 39.5% of the cases with 93.3% of the patients having long bone fractures. The images revealed 14 (93.3%) pulmonary opacities, 9 (64.3%) ground-glass opacities, 6 (42.9%) alveolar opacities, 10 (66.7%) interlobular septal thickening, and 7 pleural effusions. McDuffie et al. [14]’s study in 2022 revealed that 132 of 533 trauma cases (26.7%) had PE and 131 of 533 trauma cases (24.5%) were diagnosed with DVT. CTPA identified 21 cases (3.9%) of DVT and PE at the same time [14].

Numerous studies have examined the incidence of PE in trauma patients using CTPA, revealing significant variation in PE rates. Licht et al. (2008) reported a 12.8% incidence, while Constantino et al. (2008) [7] and Velmahos et al. (2009) [16] found rates of 9.57% and 19%, respectively. More recent research, such as Minshall et al. (2015) [15] and Buchanan et al. (2021) [9], reported PE incidences of 18% and 13%. Also, the overall prevalence of PE among the trauma patients undergoing CTPA was 18% (95%CI = 13-24%) according to our analysis. These findings underscore the substantial risk of PE in trauma patients, making it imperative to utilize timely CTPA evaluations. Effective and prompt diagnosis through CTPA is crucial for the management and treatment of PE, highlighting its vital role in improving patients’ outcomes and reducing mortality in this high-risk group.

### Clinical manifestations

The classic PE diagnostic tests at Buchanan et al. [9] study showed that the PE group had higher troponins (0.16 ± 0.22 vs. 0.06 ± 0.09, *p* = 0.04), higher rate of chest pain (19% vs. 8%, *p* = 0.04), and more prevalent leg swelling or pain (6% vs. 0.5%, *p* = 0.047); also symptoms were different among groups for instance, in contrast to non-PE patients, PE patients had shortness of breath in 26% of cases, chest pain in 19%, leg swelling and/or pain in 6%, dizziness or syncope in 16%, and hemoptysis in 0%. Vital signs were recorded differently, for example PE-patients had the vital signs as follows: temperature ≥ 101.5 F (13%), heart rate ≥ 100 bpm (77%), respiratory rate ≥ 30 rpm (39%), SBP < 90 mmHg (23%), and O2 saturation ≤ 90% (39%) [9]. Minshall et al. [15] revealed some symptoms at PE-patients group such as tachycardia (84.0%), hypoxemia (77.6%), a combination of tachycardia and hypoxia (64.8%), fever (21.2%), tachypnea (17.5%) [15]. Zhou et al. [19] found a relationship between D-dimer elevation and preoperative asymptomatic PE in patients ≥ 60 years of age with a hip fracture, but the sensitivity (82.4%) and specificity (52.1%) were relatively low [19].

The combined results of many studies on PE in trauma patients reveal a significant and variable risk associated with this condition. The high rates of PE reported earlier by Constantino et al. (2008) [7] and Velmahos et al. (2009) [16], which ranged from 9.57 to 19%, and recent studies by Minshall et al., (2015) [15] and Buchanan et al., (2021) [9] showing even higher incidence of 18–13% proves that PE is a matter of concern among these patients. This brings out the crucial importance of urgent and accurate evaluation with CTPA for quick diagnosis and effective management options which may save lives during trauma situations.

### Radiological findings

Radiologic findings at Dwivedi et al. [11] study revealed pulmonary opacity in 14 (93.3%; ground-glass opacities in 9 [64.3%], alveolar opacities in 6 [42.9%]), interlobular septal thickening in 10 (66.7%), and pleural effusions in 7 (46.7%) and filling defects were identified in three (20%) CTPAs [11]. Radiologic findings from Minshall et al. [15] study also revealed atelectasis (56%), pleural effusion (18%), and pneumonia (15%) [15]. Radiologic findings of Tofigh et al. [20] study indicated PE (26%), right and left pulmonary arteries (12%) and one side only (14%) [20]. Radiologic findings from Velmahos et al. [16] study showed 18 patients (39%) had central PE involving the main or lobar pulmonary arteries, and the remaining 28 patients (61%) had peripheral PE involving the segmental or subsegmental branches also multiple filling defects were found in 37 patients and were bilateral in 18 [16].

Various pulmonary abnormalities detected through radiological findings from multiple studies in trauma patients included opacities, effusions and embolic defects, indicating that the pulmonary problems are complex and diverse in this group. These findings show how important a comprehensive radiological evaluation is in effectively diagnosing as well as managing pulmonary issues especially in PE suspected patients among traumatic patients.

### Mortality rate

In the study by Gudipati et al. [12] 13 deaths among 85 cases of PE (15.3%), in the study by Kim et al. [4] zero cases, in the study conducted by Licht et al. [13] 3 deaths from overall 164 cases (1.8%), 1 of 7 (14.2%) late scan group (CT scans performed after 24 hours in the ICU) and 2 of 14 (14.2%) admission scan group (performed from 24 hours prior to 24 hours post ICU admission) [13], through the study done by McDuffie et al. [14] 5 deaths among 132 cases of PE (3.8%) [14], in the study by Minshall et al. [15] 17% was rate of mortality among PE positive cases [15], in the study 2 cases out of 11 cases of PE (18.1%) [15], also through study by Velmahos et al. [16] 2 cases out of 46 (4.3%) [16], and also in the study conducted by Zhou et al. [19] mortality among people with positive PE on days 30, 90, one year after diagnosis, respectively 5.8%, 11.7%, and 29.4% of total of 17 PE positive cases [19].

Mortality rates among PE cases showed considerable variability across studies, ranging from 0% to 29.4%. This underscores the importance of understanding factors influencing mortality and developing effective management protocols to mitigate risks associated with PE-related deaths.

### Quality assessment

Our quality assessment according to NOS showed that four studies [15–17, 20] had satisfactory and other ten studies had good quality (Table 2).

**Table 2:** Quality assessment of the included cohort, case-control, and cross-sectional studies

Author (ref.)	selection				comparability	outcome			Total score	Total Quality
	1	2	3	4	1	1	2	3		
Buchanan et al. [9]	*	*	*		*	*	*		6	Satisfactory
Cerbasi et al. [17]	*	*	*	*	*	*	*	*	8	Good
Costantino et al. [10]	*	*	*		*	*	*	*	8	Good
Dwivedi et al. [11]	*	*	*		*	*	*	*	7	Good
Gudipati et al. [12]	*	*	*		*	*	*	*	7	Good
Kim et al. [4]	*	*	*		*	*	*	*	7	Good
Licht et al. [13]	*	*	*		**	*	*	*	8	Good
Malinoski et al. [18]	*	*	*		**	*	*		8	Good
McDuffie et al. [14]	*	*	*		*	*	*	*	7	Good
Minshall et al. [15]	*	*	*		*	*		*	6	Satisfactory
Tofigh et al. [20]	*	*	*		*	*	*		6	Satisfactory
Velmahos et al. [16]	*	*	*		*	*	*		6	Satisfactory
Zhou et al. [19]	*	*	*		*	*	*	*	7	Good

### Publication bias

We performed Egger’s and Begg’s test to assess publication bias among the included studies which showed no evidence of publication bias (*p* = 0.98). The Begg’s funnel plot is shown in Fig. 1. Random effects model was used to account for the potential variability between studies, hence reducing the bias that could come from variance in study designs, populations and methodologies. Statistical methods were employed to determine how heterogeneous the involved studies were; heterogeneity proved substantial. This diversity was included in the analysis; thus preventing any possible biases or disparities of studies. Importantly, no evidence of publication bias was detected through statistical analyses (*p* = 0.98), implying that findings of the study are less likely to be affected by selective publication which improves its reliability.

## Discussion

According to our systematic literature review, the prevalence of PE among the wide spectrum trauma patients from traumatic fractures to trauma operation who had undergone CTPA, was 18% (95%CI = 13-24%). Interpreting these data, we should consider only a small portion of traumatized patients had undertaken CTPA; for instance, in the study by McDuffie et al. [14], out of roughly 15,000 trauma patients, approximately 500 patients were evaluated by CTPA. The mortality rate in PE patients were divergently reported from no mortality [4] to up to 29.4% [19]. This discrepancy is presumably due to the type and severity of the trauma, along with other mortality-predisposing factors such as older age, comorbid diseases, severity and extent of PE in an individual, and the treatment type or time. The discrepancy could be explained by factors such as the distribution of PE in each case and the treatment aggressiveness or time of onset.

Trauma patients are prone to develop PE. It has been suggested that trauma heightens the likelihood of VTE and accounts for around 12% of VTE cases in the population [21]. In trauma patients, several factors including paralysis-immobilization, endothelium damage/venous-trauma, hypercoagulability, blood transfusion, and inflammation increase the possibility of PE [22]. PE has been suggested to be one of the potentially preventable causes of death in severely traumatized patients being responsible for roughly 12% of all deaths [23]. PE is a significant avoidable factor leading to mortality in seriously injured patients, accounting for around 12% of all fatalities. Timely identification of PE following trauma greatly reduces the chance of death and lowers the probability of enduring sequelae. To achieve this goal, CTPA has significantly enhanced the diagnosis of PE and is regarded as the standard imaging test [24, 25].

CTPA has a sensitivity of 96–100% and a specificity of 89–98% in diagnosing probable PE [26]. CTPA has several significant benefits that contribute to its general acceptance. The main advantages of this procedure are its accessibility, little invasiveness, and rapidimaging time [27]. CTPA’s field of view extends beyond just the pulmonary arteries, which allows the visualization of various causes of shortness of breath and chest pain, including musculoskeletal injuries, pneumonia, pericardial abnormalities, and vascular pathologies [28]. Moreover, if a CTPA is normal in patients with low or intermediate clinical likelihood, the diagnosis of PE can be excluded without further testing. Stepping outside of its benefits we should consider that excessive testing for PE is a significant health issue, particularly at university hospitals [29, 30]. At one center, if the Revised Geneva Score was used, an estimated 9.84% of CTPA could have been avoided [31]. Although improvements in procedures and techniques can optimize diagnostic accuracy while reducing radiation exposure, the primary issue associated with utilizing CT scans is the potential danger of cancer due to ionizing radiation, particularly in young female patients or patients that are frequently seen for recurrent chest pain [32, 33]. Nonetheless, Woo et al. demonstrated a notable benefit-to-risk ratio of CTPA by considering the mean lifetime attributable risk of cancer death [34]. Furthermore, CTPA is conducted using intravenous contrast agents, which can lead to contrast-induced nephropathy, but is much less common than previously thought, but can rarely happen with impaired renal function [35, 36]. For current low-osmolar and iso-osmolar contrast materials, the risk of other adverse events, associated with intravenous iodinated contrast is minimal, falling between 0.2% and 0.7% [37].

Regarding the limited resources and complications CTPA continues to be the gold standard of diagnosing PE [38], it is suggested to be employed for highly suspected patients characterized with relevant clinical features and laboratory findings. The usual procedure for a suspected PE in a non-traumatized patient is to exclude PE using either subjective clinical judgment or objective criteria, such as a risk score model. According to society standards for both emergency medicine and internal medicine, if the pretest likelihood of a PE is minimal and accompanied by a normal D-dimer, PE may be ruled out [39, 40]. In trauma patients, the significance of a D-dimer in diagnosing pulmonary embolism is uncertain because traumatic damage can generate increased D-dimers regardless of the presence of a pulmonary embolism [41, 42]. One of the most well-known clinical-based scoring systems to assess the probability of PE is Wells criteria. As shown by Buchanan et al. [9], although among the current PE-predicting risk scoring models, employing Wells criteria in trauma patients appeared to have the highest area under the curve of 0.65, this criteria failed to predict the PE optimally. In addition, on a population of patients with femur fractures, simplified Wells score showed no significant difference between PE and non-PE groups [4]. These findings are in contrast to the results of recent meta-analysis of 8947 patients which showed acceptable capability of Wells and Geneva rules as well as D-dimer level to diagnose PE [43]. It should be noted that the meta-analysis findings are not limited to trauma patients, which may be a confounded factor for this discrepancy. The classic signs and symptoms for PE may be masked in trauma patients by other disease processes that causes hypoxemia, tachypnea, tachycardia and dyspnea [5, 6]. Approximately 29% of post-trauma PE occurs within the first four days following the trauma, of which long bone fractures and chest injuries were the most associated injuries with the occurrence of 70.8% and 45.8%, respectively [44]. Hence, in evaluating the trauma patients in order to screen PE, we should not solely rely on classic manifestation of the disease.

### Limitations

This systematic review and meta-analysis encounters several potential limitations. First, we were unable to clarify the impact of the severity of trauma, the specific location of fractures, and the presence of comorbid conditions on the incidence of PE in trauma patients. Such approach is crucial for understanding the PE risk factors and could significantly enhance individualized patient risk stratification and management strategies.

Secondly, the included literature spans a broad spectrum of trauma types, ranging from polytrauma to elective operations. This heterogeneity represents a challenge in discriminating the specific risk factors and mechanisms of PE development unique to each trauma subtype. Our analysis did not segregate these groups distinctly, potentially obscuring the varied etiologies and risks associated with different types of traumas. This limitation highlights the need for more targeted studies that can dissect the relationship between specific trauma types and the risk of PE.

Thirdly, although some studies reported the interval time between trauma occurrence and PE diagnosis through CTPA, there was not sufficient data to analyze their reports systematically. This temporal relationship is critical for understanding the timing of PE development post-trauma and could inform both surveillance strategies and the timing of diagnostic testing. The absence of this data limits our ability to recommend optimal timing for PE screening in trauma patients, which could potentially affect patient outcomes.

Another limitation of our study was high heterogeneity in the result of meta-analysis calculating the pooled prevalence of PE among trauma patients undergone CTPA. Also, subgroup analysis and meta-regression were not applicable due to variety of the included studies in reporting data. Therefore, the results of meta-analysis should be interpreted with caution.

Finally, it’s important to acknowledge that while our systematic review and meta-analysis provides valuable insights into the prevalence and significance of PE in trauma patients with CTPA evaluation, it’s not standard practice for all trauma patients to receive CTPA studies. Therefore, the generalizability of our findings to all trauma populations may be limited. The decision to perform CTPA in trauma patients often depends on various factors, including the severity and mechanism of injury, clinical suspicion for PE, and availability of resources. Thus, the prevalence rates of PE reported in our study may not fully represent the entire spectrum of trauma patients.

## Conclusion

In conclusion, our systematic review and meta-analysis demonstrates CTPA’s crucial significance in identifying PE in trauma patients, highlighting its high sensitivity and specificity. Despite its effectiveness, the diversity in PE frequency among trauma patients, as well as the accompanying death rates, highlight the challenges of identifying and treating PE in this group. The limits of D-dimer testing and clinical prediction guidelines in the trauma population, together with the confounding effects of trauma, demand a careful approach to diagnosing PE. This study emphasizes the importance of CTPA in traumatic situations to diagnose, improving treatment, weighing the advantages of early and accurate PE diagnosis against the hazards and costs of CTPA. According to our findings, lower threshold for CTPA is recommended in patients with lower extremity or spine fractures. Future research should aim to precisely demonstrate the differences between trauma and other medial nature of PE development and subsequently modify the guidelines specified for trauma patients refine diagnostic strategies.

## Data Availability

All figures and tables are available in the main paper.
